# LED Light-Quality Optimization to Enhance Shoot and Essential Oil Yield of *Tagetes erecta* L. in Controlled Environment

**DOI:** 10.3390/molecules31091491

**Published:** 2026-04-29

**Authors:** Ha Thi Thu Chu, Nhung Hong Nguyen, Quyen Phan, Thuy Thi Thu Dinh, Trang Huyen Thi Hoang, Tru Van Nguyen, Ha Hoang Chu, Quang Cong Tong, Tran Quoc Tien, William N. Setzer, Khanh Quoc Tran, Phat Tien Do

**Affiliations:** 1 Institute of Biology, Vietnam Academy of Science and Technology (VAST), 18 Hoang Quoc Viet, Nghia Do, Ha Noi 10072, Vietnam; cttha@ib.vast.vn (H.T.T.C.); nhungnhn93@gmail.com (N.H.N.); phanquyen782@gmail.com (Q.P.); hhtrang@ib.ac.vn (T.H.T.H.); nvtru@ib.ac.vn (T.V.N.); chuhoangha@ib.ac.vn (H.H.C.); 2 Institute of Chemistry, Vietnam Academy of Science and Technology, 18 Hoang Quoc Viet, Nghia Do, Ha Noi 10072, Vietnam; dttthuy@ich.vast.vn; 3 Institute of Materials Science, Vietnam Academy of Science and Technology, 18 Hoang Quoc Viet, Nghia Do, Ha Noi 10072, Vietnam; congtq@ims.vast.ac.vn (Q.C.T.); tientq@ims.vast.ac.vn (T.Q.T.); 4 Aromatic Plant Research Center, 230 N 1200 E, Suite 100, Lehi, UT 84043, USA; set-zerw@uah.edu; 5 Department of Chemistry, University of Alabama in Huntsville, Huntsville, AL 35899, USA; 6 Laboratory of Adaptive Lighting Systems and Visual Processing, Technical University of Darmstadt, Hochschulstr. 4a, 64289 Darmstadt, Germany

**Keywords:** African marigold, antimicrobial activity, (*E*)-*β*-ocimene, (*E*)-myroxide, LED light spectra, piperitone

## Abstract

This study evaluated the effects of light spectral quality on shoot yield and essential oil of *Tagetes erecta* L. cultivated in controlled growth chambers under three LED lighting treatments with different red, blue, and white wavelength ratios and a constant 16 h photoperiod for up to 101 days. The F2 treatment (5 red:1 blue) produced yields of fresh shoots, early blooming flowers, and oils of 1586 ± 164 g/m^2^, 569.77 ± 76.81 g/m^2^, and 307 ± 31.7 mg/m^2^, respectively. These values were significantly higher (*p* < 0.05) than those of the F1 treatment (white:red-phosphor), and represented increases of 1.37-, 1.26-, and 1.38-fold, respectively. Gas chromatography identified 30–31 compounds in the oil with three major constituents—(*E*)-*β*-ocimene (22.9–28.8%, highest under F3), (*E*)-myroxide (13.9–20.6%, highest under F1), and piperitone (7.3–9.6%, highest under F3). Essential oils inhibited from four to five of the seven tested microbial strains, with the notable activity against *Escherichia coli* and *Candida albicans* recorded in F2 and F1, respectively. These findings confirm that light spectral quality is a critical factor regulating flower, essential oil, and antimicrobial efficacy in *T. erecta*, demonstrating that optimized LED spectra offer a practical strategy to improve plant yield and phytochemical quality.

## 1. Introduction

African marigold (*Tagetes erecta* L.), a member of the Asteraceae family, is an annual herbaceous species characterized by pinnately compound leaves, and large flowers—either single or double capitula that range from bright yellow to deep orange. Owing to its prolific flowering, vivid coloration, and relatively short growth cycle, the African marigold is widely cultivated as an ornamental crop. Among six evaluated *Tagetes* species, African marigold has demonstrated the highest flower yield, reaching up to 7.3 t ha^−1^ [1]. Species of the genus *Tagetes* are well known for their insecticidal and nematicidal properties [2,3]. They are frequently incorporated into crop rotations as natural pest-repellent plants due to the bioactive volatile compounds present in their leaves and flowers. The flowers and their extracts are used in traditional medicine for anti-inflammatory, antiseptic, and digestive purposes. In addition, its antibacterial and antioxidant activities were investigated [4]. On the other hand, the flowers are rich in carotenoids, particularly lutein, which are extracted for use as natural food colorants, feed additives, and ingredients in pharmaceutical and cosmetic formulations [5]. Essential oils from *Tagetes* species are valued in perfumery, deodorants, and personal care products, and are increasingly studied for applications as biopesticides and flavoring agents [6]. Species such as *T. minuta* and *T. erecta* supply essential oils to niche segments of the fragrance industry. However, essential oil content and composition vary widely depending on plant organ, cultivar, geographic origin, environmental conditions (including light and nutrition), harvest time, and extraction method [7,8,9,10,11,12]. Although African marigold holds substantial medicinal and economic value and is widely cultivated commercially, such compositional variability poses challenges for product standardization, underscoring the need for optimized and standardized cultivation, harvesting, drying, and extraction protocols.

African marigolds can be cultivated and harvested under traditional open-field conditions; however, greenhouse production enables year-round cultivation and greater environmental control. In such systems, optimizing growth conditions—particularly light supply—is essential to ensure high productivity and consistent product quality. Light is a key ecological factor regulating the growth, morphology, and physiology of Asteraceae in general and *Tagetes* species in particular. Beyond serving as the primary energy source for photosynthesis, light acts as a regulatory signal influencing flowering, branching, pigment synthesis, and essential oil biosynthesis [11]. Both light intensity and spectral quality—especially within the red (R), blue (B), green (G), and far red (Fr) regions—strongly affect the biosynthesis and accumulation of secondary metabolites such as carotenoids, flavonoids, phenolics, and terpenoids, which determine the aroma, bioactivity, and medicinal value of this species [13]. R and B wavelengths regulate stem elongation, leaf expansion, and photosynthetic enzyme activity [14], while irradiance, spectrum, and photoperiod collectively modulate gene expression associated with secondary metabolic pathways. Spectral variation can shift plant resource allocation between biomass production and secondary compound accumulation [15]. Numerous studies highlight the importance of optimizing R:B ratios. For example, R:B (1:1) increased fruit size and weight in chili pepper (*Capsicum annuum* ‘Cheonyang’) while enabling control of capsaicinoid and carotenoid content [16]. In strawberry (*Fragaria* × *ananassa*), R:B (1.1:1.0) improved fresh weight compared with white (W) light and other R:B ratios [17]. Various R:B combinations also enhanced vegetative and floral traits in *Lilium brownii* var. *viridulum* ‘Corvara’ [18]. In *T. minuta*, moderate shading (~25%) increased total oil content relative to full sun or heavy shade, whereas 50–70% shading reduced oil yield and altered composition [11]. LED studies in *T. erecta* showed that combined R:B (1:1) lighting or cool white fluorescent lamps at 90 ± 10 µmol m^−2^ s^−1^ under a 16 h photoperiod enhanced dry biomass accumulation, while R:Fr (1:1) increased bud formation, and fluorescent light maximized open flower number [19]. A recent study reported that R-light increased root biomass and leaf area, promoting vegetative growth in *T. erecta*, while B-light enhanced chlorophyll fluorescence and photosynthetic efficiency but contributed less to biomass accumulation. The combination of R and B-light produced optimal photosynthetic performance [20]. R-light generally promotes early flowering and biomass production, whereas higher B fraction often enhances plant height, pigment accumulation, antioxidant activity, and certain secondary metabolites. Thus, the R:B ratio should be tailored according to production objectives, whether maximizing flower and essential oil yield or enhancing phytochemical quality [21]. Furthermore, a daily light integral (DLI) ≥ 10 mol m^−2^ d^−1^ during the finishing stage was shown to produce uniformly high-quality *T. erecta* stems [22]. Spectral management is also important during post-production: fluorescent or green LED lighting effectively maintained the quality of *T. erecta* ‘Orange Boy’ seedlings during 28 days of storage at 8 °C under low irradiance conditions [23].

Studies employing monochromatic or combined narrow-band R and B LEDs have received considerable attention. However, investigations into the effects of multispectral lighting on plants—particularly on *T. erecta*—remain limited. In particular, there is a notable lack of data on how varying R-to-B LED ratios, as well as multispectral LED systems, influence the yield, chemical composition, and antimicrobial activity of essential oils in *T. erecta*. To better simulate realistic multiwavelength conditions and optimize both growth and product quality of plants, this study focuses on light treatments with varying R:B ratios supplemented with a small proportion of W light.

Optimizing artificial light spectra represents a promising strategy for enhancing growth and increasing the biological value of Asteraceae species by regulating the accumulation of bioactive secondary metabolites. This approach is particularly important for medicinal and essential oil crops, in addition to supporting the ornamental value of Asteraceae in general and African marigold in particular. Cultivation of medicinal plants in controlled growth chambers—where temperature, humidity, and light spectra are precisely regulated—offers significant advantages in the context of climate change. Such systems buffer crops from heat stress, heavy rainfall, and extreme weather fluctuations, ensuring uniform growth and potentially shortening production cycles. Spectral and irradiance optimization enhances photosynthetic efficiency and stimulates the biosynthesis of biologically active secondary compounds, which are critical determinants of medicinal quality. Precise environmental control also reduces pest and disease pressure, minimizes pesticide use and water consumption, and facilitates the production of clean, standardized plant materials. Moreover, controlled environments enable year-round and off-season cultivation, ensuring a stable supply chain for medicinal and ornamental markets. Collectively, this production model enhances yield, appearance, and phytochemical quality while supporting sustainable and climate-resilient agriculture [24,25,26].

Building on these advantages, the present study evaluated the effects of different light spectra on the growth, development, and essential oil accumulation of *T. erecta* under controlled growth chamber conditions. Specifically, three lighting treatments were designed in this study. The W + R-phosphor treatment (F1) represents commercially available white LED systems commonly used in greenhouse production. The R:B ratio of 5:1 (F2) was selected based on previous studies indicating that R-dominant spectra promote biomass accumulation and flowering. The 3R:2B:1W combination (F3) was included as a more balanced spectrum, incorporating B-light to support secondary metabolism and a white component (including G wavelength) to enhance spectral completeness and better simulate practical horticultural lighting conditions. By systematically adjusting spectral composition, we aimed to identify optimal wavelength combinations that promote uniform vegetative growth, synchronized flowering, and enhanced synthesis of bioactive secondary metabolites. Such optimization not only improves medicinal quality and supply reliability but also enhances ornamental traits, including flower size and morphology. To our knowledge, this is the first study to comprehensively assess the impact of multispectral LED lighting on essential oil content, chemical composition, and antimicrobial activity in *T. erecta*, providing new insights for sustainable and precision-based cultivation strategies.

## 2. Results

### 2.1. The Effect of LED Light Conditions on Biomass and Essential Oil Yield of Tagetes erecta

**Figure 1:**
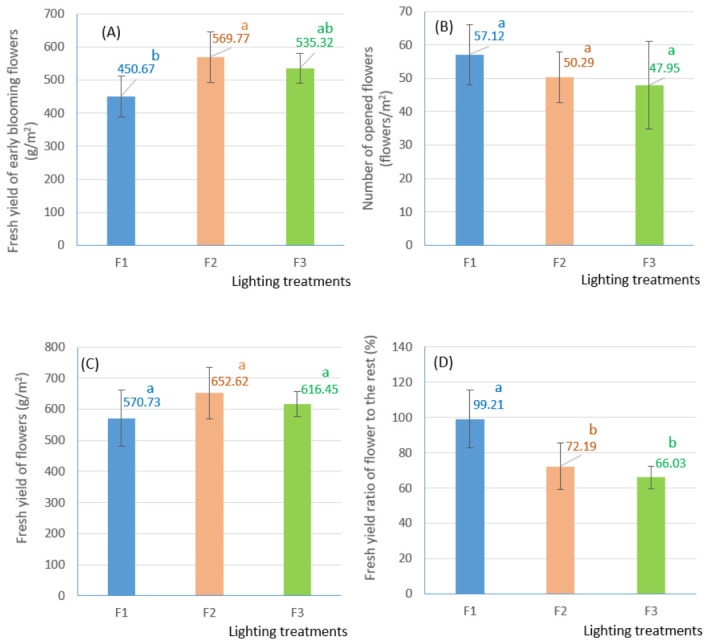
Some physiological parameters of *Tagetes erecta* cultivated under different light conditions. (**A**) Fresh yield of early blooming flowers; (**B**) Number of opened flowers; (**C**) Fresh yield of flowers; (**D**) Fresh yield ratio of flowers to the rest. (Note: Mean values followed by the same letter within a chart are not statistically different at a 0.05 significance level (*n* = 6). Statistical analyses were performed using IRRISTAT ver. 5.0 (International Rice Research Institute, Laguna, Philippines)).

The different light spectra had distinct effects on growth, development, and essential oil biosynthesis in the aerial parts of *Tagetes erecta* L. (African marigold). The time to first flowering (defined as at least one fully opened flower per plant) ranged from 11 weeks to more than 14 weeks. White LEDs coated with red-emitting phosphor (WRp, treatment F1) promoted earlier flowering (77 days) compared to treatments combining R with B and/or W lights (91 and 101 days in treatments F2 and F3). A significant difference (*p* < 0.05) in fresh yield of the first-opened flowers was observed between F2 (5R1B, 569.77 ± 76.81 g/m^2^) and F1 (WRp, 450.67 ± 61.37 g/m^2^). In contrast, the value in F3 (3R2B1W, 535.32 ± 44.59 g/m^2^) did not differ significantly from either treatment (Figure 1A). At harvest, each plant yielded 1–2 opened flowers, with the highest average number of opened flowers obtained in F1 (57.12 ± 9.04 flowers/m^2^). The lowest number was recorded in F3 (47.95 ± 13.02 flowers/m^2^), but the difference was not significant between the treatments (Figure 1B). The fresh yields of total flowers were also slightly different between the lighting treatments, which ranged from 570.73 ± 90.48 to 652.62 ± 82.87 g/m^2^ (Figure 1C). The proportion of flower fresh yield relative to the combined yield of stems, leaves, and buds varied between 66.03% ± 6.32 and 99.21 ± 16.29%. The F1 yielded the highest flower weight ratio, which differed significantly (*p* = 0.001) from the F2 and F3 treatments (Figure 1D).

The fresh yield of shoot parts of the African marigold was highest under treatment of F2 (1586 ± 164 g/m^2^), followed by F3 (1568 ± 123 g/m^2^). These values were significantly higher (*p* < 0.001) than the treatment of F1 (1157 ± 170 g/m^2^). Plant water content ranged from 89.2 to 90.2%, with the lowest value achieved in treatment F1. Dry yields of the shoot part were greatest under treatments F3 (157 ± 12.3 g/m^2^) and F2 (155 ± 16.0 g/m^2^), with no significant difference between them. Both values were significantly higher (*p* = 0.003) than that of treatment F1 (125 ± 18.4 g/m^2^). Essential oil concentration and yield differed significantly (*p* < 0.001) among the three treatments, with the highest value observed in F2 (0.2285 ± 0.0155% and 307 ± 31.7 mg/m^2^), followed by F3 (0.1974 ± 0.0126% and 252 ± 19.8 mg/m^2^), and the lowest value in F1 (0.1541 ± 0.0101% and 222 ± 32.7 mg/m^2^) (Table 1).

### 2.2. The Effect of LED Light Conditions on Essential Oil Composition of Tagetes erecta

The essential oils extracted from the aerial parts of the African marigold under different light treatments contained 30–31 identified compounds, representing 93.7–94.7% of the total composition. The predominant groups were monoterpene hydrocarbons (40.2–46.8%) and oxygenated monoterpenes (35.8–42.0%). Three major constituents were identified: (*E*)-*β*-ocimene (22.9–28.8%), (*E*)-myroxide (13.9–20.6%), and piperitone (7.3–9.6%). The contents of each of these three main compounds in African marigold essential oil differed significantly (*p* < 0.001) among the three light treatments in the current study. The highest concentrations of (*E*)-*β*-ocimene and piperitone were observed under illumination treatment F3, whereas the lowest were recorded under F1. In contrast, (*E*)-myroxide reached its highest concentration under F1 and its lowest under F3. Other relatively abundant constituents included limonene (5.2–5.7%), terpinolene (4.5–5.5%), 3-thujyl acetate (4.0–5.1%), and piperitenone (4.3–6.2%). The remaining compounds had concentrations ranging from trace (Tr) to 3.8% of the total oils (Table 2).

### 2.3. The Effect of LED Light Conditions on Antimicrobial Activity of the Essential Oil of Tagetes erecta

The antimicrobial activity of African marigold essential oils was tested against seven microbial strains: three Gram-positive bacteria (*Staphylococcus aureus*, *Bacillus subtilis*, and *Lactobacillus fermentum*), three Gram-negative bacteria (*Salmonella enterica*, *Escherichia coli*, and *Pseudomonas aeruginosa*), and one yeast (*Candida albicans*). Overall, the African marigold essential oils grown under different lighting conditions showed no inhibitory effect against *B. subtilis*. Another common characteristic of these samples is their inhibition against three pathogenic microorganism strains, including *S. aureus*, *E. coli*, and *P. aeruginosa*. The MIC values of all three oil samples against the seven tested microorganism strains ranged from 8000 to >16,000 µg/mL. The oil from treatment F1 demonstrated the strongest antimicrobial activity, inhibiting 5/7 strains. It was most effective against *S. aureus*, *S. enterica*, and *C. albicans* (IC_50_ = 4500 ± 201 to 13,818 ± 610 µg/mL). The oil from treatment F2 also inhibited 5/7 strains, with IC_50_ values of 4758 ± 225 to 12,000 ± 579 µg/mL. Notably, it inhibited *E. coli* most strongly compared with other treatments. By comparison, F3 inhibited 4/7 strains, with IC_50_ values ranging from 7571 ± 377 to 16,000 ± 577 µg/mL, indicating the weaker inhibition (Table 3).

## 3. Discussion

In higher vascular plants, radiation is perceived by several classes of photoreceptors. Phytochromes primarily absorb R (601–700 nm) and Fr (701–800 nm) wavelengths, cryptochromes absorb B (400–500 nm) and UV-A radiation, and phototropins are specialized B-light receptors, as first characterized in *Arabidopsis thaliana* [27]. According to earlier studies, these photoreceptors interact to regulate flowering, photomorphogenesis, and photosynthetic development [28,29,30]. In the present study, treatment F1 (WRp) provided broad spectral compositions, thereby promoting earlier reproductive development [31]. Notably, this treatment contained a higher proportion of Fr radiation (12.45%, a key signal for floral induction mediated primarily through phytochromes [32,33]. Phytochromes exist in two interconvertible forms: the inactive Pr (R-absorbing) and the active Pfr (Fr-absorbing) form, with their relative abundance regulated by R and Fr radiation [34]. Fr enrichment can accelerate flowering by reducing the level of active phytochrome B (Pfr), a known floral repressor in both long-day and short-day species [35,36]. Earlier flowering under R:Fr (1:1) irradiation was also reported in marigold at 90 ± 10 µmol m^−2^ s^−1^ [19]. In contrast, F3 (3R2W1B) treatment exhibited the latest flowering time (*p* < 0.001), possibly due to its higher proportions of B-light (42.05%) and G-light (10.22%)which can delay flowering [37,38]. B-light at ≥20 µmol m^−2^ s^−1^ promoted flowering in long-day plants but might inhibit flowering in short-day species when applied as night interruption or photoperiod extension [39,40], whereas the lower B proportion in F1 treatment was insufficient to delay flowering, which is consistent with reports that low-intensity nocturnal B-light does not affect flowering in some short-day plants, including African marigold [41]. A noteworthy finding of this study is that the time to first flowering is inversely proportional to the intensity ratio of R- to B-light. This observation aligns with previous research demonstrating that R-light plays a key role in promoting earlier flowering in African marigold [21]. Collectively, these results highlight the central role of phytochrome-mediated signaling in regulating flowering and demonstrate how spectral adjustment can be used to control vegetative growth and reproductive timing in a species-specific manner.

The greatest fresh yield of early-blooming flowers, achieved under the treatment with the highest fractions of R- and B-light (73.45%:25.82%, F2), underscores the synergistic role of these two wavelengths in promoting floral biomass during African marigold growth and development. Chlorophyll exhibits absorption peaks around 430 nm and 660 nm, corresponding to B and R-light, respectively; therefore, these wavelengths are particularly effective in driving photosynthesis and biomass accumulation [42]. The promotive effects of R- and B-light observed in this study are consistent with previous reports. R-light was shown to significantly increase biomass, while B-light enhances plant height in African marigolds [43]. A 3:1 R-to-B-LED ratio supported morphological development and increased average fruit weight in tomato (*Solanum lycopersicum*) [44]. Similarly, a 7:1 ratio improved dry weight in Chinese cabbage (*Brassica chinensis*) compared with HPS lighting [45], and 8:1–9:1 ratios enhanced shoot biomass in cucumber (*Cucumis sativus*) [46]. These improvements are likely associated with enhanced photosynthetic capacity and increased stomatal conductance under combined R- and B-lights, facilitating CO_2_ assimilation and transpiration [14]. However, plant responses are strongly ratio-dependent. In tomato, R:B (9:1) increased dry weight more than 7:3 or 1:1 ratios [47], whereas fruit yield improved only under R:B (5:1) at 135 μmol m^−2^ s^−1^ [48].

The lowest yield of early-blooming flowers was observed under the treatment of F1, likely because flowering occurred too early, before the plants had reached peak vegetative growth and accumulated sufficient biomass, thereby limiting flower development and quality. In addition, the presence of a G-light component in F1 (5.88%) may have further contributed to the reduced flower yield. Although treatment F3 flowered later than F2 (101 days versus 91 days), its higher proportion of G-light (10.22% versus 0.52%) may have negatively influenced assimilate accumulation, resulting in a slightly lower flower yield compared with F2. This is consistent with previous research showing the negative impact of G-light on the biomass of tomato and basil [49].

The highest flower number observed in F1 aligns with studies showing that fluorescent light supplemented with R-LED enhances flowering in African marigold [19,43]. Likewise, R:B (3:1) increased flower number by 15% compared with R:B (1:1) or HPS lighting under continuous supplemental illumination [50], and similar results were reported for the ‘Antigua Orange’ cultivar at 120 μmol m^−2^ s^−1^ [20]. A comparison of the data recorded between the two treatments: F2 (R:B = 73.45%:25.82%) and F3 (R:B = 47.30%:42.05%) in the current study is also consistent with this trend. Conversely, while the F1 treatment showed the highest number of African marigold flowers, its fresh flower yield was the lowest. This indicates a smaller flower size compared to the other treatments. On the other hand, the ratio of flower yield to the yield of the rest of the shoot was highest in F1, meaning that the stem, leaves, and flower buds of the marigolds also had the lowest biomass among the treatments.

The higher fresh yield of shoot parts of African marigold under F2 and F3 compared to the one under treatment F1 indicates that LED illuminations containing R and B spectra at the ratios of 2.84:1 (73.45%:25.82%) and 1.12:1 (47.30%:42.05%) have more positive effects on the growth of this species than the ratio of 3.71:1 (64.28%:17.33%). Simultaneously, these results further demonstrate that the precise balance of R and B spectral components, unique to each species and variety, is critical for optimal plant development. In addition, the G-depleted spectrum in treatment F2 (0.52%) likely promoted plant growth, resulting in greater biomass accumulation compared with treatments containing higher proportions of G-light. This result is consistent with earlier findings reported by Klein et al. [51].

The lower value of plant water content achieved in treatment F1 may be due to the impact of the high ratio of R:B and the high proportion of Fr in F1, through a mechanism of increased stomatal conductance leading to increased transpiration [52,53].

The effect of light on the shoot yield of African marigold showed the most positive response under the F2 treatment in the present study. This result is relatively consistent with previous research showing that a B:R (1:3) spectral ratio under supplemental lighting increased above-ground biomass yield in African marigold, compared with a B:R (1:1) and high-pressure sodium (HPS) lighting [50]. The essential oil content observed in this study was also comparable to previous reports, e.g., floral essential oil reached up to 2.5% of fresh weight [1], while leaf essential oil content was approximately 0.2% [54]. Variation in oil content in African marigold appears to be influenced, at least in part, by spectral quality. Under equivalent light intensities in the present study, treatments containing a high total fraction of R- and B-lights (treatments F2, R + B = 99.27%, and F3, R + B = 89.35%) significantly enhanced essential oil biosynthesis and accumulation compared with the WRp treatment (F1, R + B = 81.61%). These results highlight the positive role of combined R- and B-wavelengths in stimulating secondary metabolite production, consistent with findings in lettuce [55]. A previous study demonstrated that R-light positively influenced essential oil production in *Thymus migricus* and *T. carmanicus*, whereas W-light exerts a stimulatory effect in *T. vulgaris* and *T. kotschyanus* [56]. In general, plant responses to light spectra are species-specific, resulting in considerable variation in the accumulation of volatile compounds. To our knowledge, no previous studies have specifically examined the effects of light spectral composition on essential oil accumulation in African marigold, underscoring the novelty of the present work.

Thus, these results demonstrate that different light spectra distinctly influence the growth of African marigolds. Therefore, light wavelength composition can be strategically adjusted to regulate plant photomorphogenesis under indoor cultivation, depending on production goals. To promote early flowering and increase flower number, incorporating a small proportion of Fr-light into the growing environment is recommended. On the other hand, to achieve larger flower size, higher shoot yield, and greater accumulation of volatile compounds, cultivation conditions should minimize or exclude G-light and maintain an R:B ratio between 1.12:1 and 2.84:1, rather than using a higher ratio such as 3.71:1. These recommendations apply under conditions of light intensity and other environmental parameters equivalent to those established in this study.

Correlation analyses were implemented to examine (i) the relationship between the R:B ratio and essential oil concentration, and (ii) the association between (*E*)-*β*-ocimene concentration and antimicrobial activity. Pearson correlation coefficients (r) were performed using SPSS ver. 27.0.1 (IBM SPSS Statistics). African marigold essential oil concentration showed a negative correlation with the R:B ratio (r = −0.418). In contrast, the (*E*)-*β*-ocimene concentration was positively correlated with antimicrobial activity against five of the seven tested microbial strains. The strongest correlation (r) was observed for the Gram-negative bacteria *S. enterica* (0.970), followed by *P. aeruginosa* (0.749), *C. albicans* (0.711), and *S. aureus* (0.704). No correlation was found between (*E*)-*β*-ocimene content and activity against *B. subtilis*. Furthermore, the essential oil content showed a statistically significant correlation (*p* < 0.05) with antimicrobial activity against *P. aeruginosa* (r = 0.999) (Table 4).

The essential oil composition of the African marigolds reported in this study is not much different from findings of previous research, although relative proportions vary. For example, flower and leaf samples from Italy contained limonene (3.5% and 15.6%), terpinolene (5.8% and 28.5%), and piperitone (28.9% and 24.2%) [1]. In Brazil, leaf samples were rich in terpinolene (12.4%), (*E*)-*β*-ocimene (13.1%), piperitone (20.0%), and limonene (11.0%) [54]; and limonene (10.4%), dihydrotagetone (11.8%), α-terpinolene (18.1%), and (*E*)-*β*-ocimene (13.0%) [57]. Samples from India showed limonene (7.6% and 6.9%), terpinolene (11.2% and 4.7%), (*Z*)-myroxide (4.2% and 7.9%), piperitone (52.4% and 28.5%), piperitenone (5.0 and 10.9), and varying levels of piperitenone oxide, and β-caryophyllene [58]. Flowers generally produce higher levels of oxygenated terpenes than leaves [12,59]. The data of African marigold essential oils show recurring major constituents, but their relative abundance depends on cultivar, plant part, environmental conditions, developmental stage, extraction method, and geographic origin. Notably, the essential oil sample in this study had significantly higher levels of (*E*)-*β*-ocimene and (*E*)-myroxide compared to samples from other countries. Lighting conditions with a high R:B ratio increased the accumulation of (*E*)-myroxide, while the opposite effect was observed for the most dominant compound, (*E*)-*β*-ocimene. The study results showed that the synergistic effect of these primary spectra (R and B), when applied at appropriate ratios and intensities, not only enhances plant growth but also actively promotes the accumulation of the target compounds. Depending on the intended post-harvest use of African marigolds, specific spectral conditions can be selected. For instance, to maximize (*E*)-*β*-ocimene yield, an R:B ratio of 1.12:1 under comparable environmental conditions may be applied. In addition, Fr and G spectral components may also partly contribute to this effect; however, this aspect has not yet been systematically investigated.

The African marigold essential oil from treatment F1, with the highest proportion of oxygenated monoterpene group (42.0%), may be the reason for its leading antimicrobial activity against three strains: *S. aureus*, *S. enterica*, and *C. albicans*. However, the inhibition levels of African marigold essential oils against other microbial strains may be due to the synergistic effect of their constituents. The essential oil of African marigold leaves contains high levels of (*Z*)-*β*-ocimene, which has been shown to have antifungal activity against several pathogenic fungi, with IC_50_ values from Petri dishes of 2–6 µL/80 mm depending on the fungal strains tested [60]. Some previous research showed that the dominance of tagetone and ocimene contributes to the characteristic aroma and reinforces the broad spectrum of bioactivity associated with African marigold essential oil [6,12]. Overall, African marigold essential oil in different light formulations showed inhibitory activity against four to five out of seven tested microorganism strains. None of the oil samples inhibited *B. subtilis*, and only one oil sample weakly inhibited *L. fermentum*. Previous studies indicated that African marigold essential oil exhibits very low or no toxicity in animal models. Specifically, weak toxicity was observed in mice [61], and dermal exposure on rats produced no toxic effects [62]. In contrast, in vitro studies reported high cytotoxic activity of African marigold essential oil against both tumor and normal cell lines [57,63]. This is the basis for the potential use of African marigold essential oil in serving human life activities.

These findings indicate that the antimicrobial activity of African marigold essential oil can be modulated by altering light spectral composition during cultivation. WRp LED lighting under the conditions established in this study can be recommended, as it inhibited five harmful microorganisms while showing no inhibitory effects on two beneficial Gram-positive bacteria.

**Figure 2:**
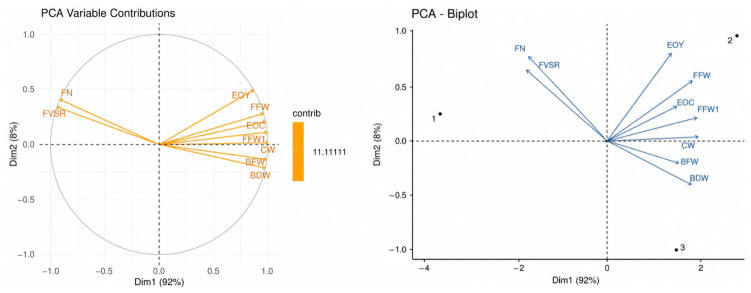
PCA of treatment effects and key contributors in *Tagetes erecta*. Note: PAC analysis was performed using software PCA & HCPC Analysis ver.1.0—developed by the Vietnamese Association at Kyushu University (Nguyen, Trung Duc, https://ntducphd.shinyapps.io/pca_plus/; accessed on 22 April 2026); BFW = Fresh yield of shoot (g/m^2^); CW = Water content (%); BDW = Dry yield of shoot (g/m^2^); EOC = Essential oil content (% *w*/*w*, dry); EOY = Essential oil yield (mg/m^2^); FFW1 = Fresh yield of early blooming flowers (g/m^2^); FN = Number of opened flowers (flowers/m^2^); FFW = Fresh yield of flowers (g/m^2^); FVSR = Fresh yield ratio of flowers to the rest (%).

To clarify overall trends in the dataset and highlight differences among treatments, as well as the main factors contributing to biomass and essential oil yield, the tool of principal component analysis (PCA) was applied to reduce data complexity. The results are presented in Figure 2.

PCA revealed that plants producing many flowers (high FN and FVSR) generally showed lower overall yield (low EOY, FFW, and BFW). Conversely, plants with greater biomass accumulation and yield (high EOY, FFW, and BFW) tended to produce fewer flowers. This pattern indicates a clear antagonistic relationship between reproductive output and biomass-related traits, primarily along PC1, which explained 92% of the total variation. Based on the PCA plot, the three samples can be characterized as follows:

Sample 1 (F1): Associated with high flower number but low cumulative yield.

Sample 2 (F2): Characterized by high flower yield and elevated essential oil content.

Sample 3 (F3): Associated with strong biomass accumulation (vigorous stem and leaf growth) but moderate flower and essential oil yield.

## 4. Materials and Methods

### 4.1. Plant Materials and Lighting Conditions

The experiment was conducted in a growth chamber in Hanoi, Vietnam (N21°04′07″, E105°45′51″). The seeds of *Tagetes erecta* L. (African marigold), cultivar PH-10, purchased from Phuong Hoang Ltd. company in Ho Chi Minh city, Vietnam, were sown in February 2024. The seedlings were transplanted in March 2024 with a distance of 20 × 15 cm, six plants per one black plastic rectangle tray (38 × 45 × 12 cm). A substrate mixture of peat moss, vermiculite, and perlite at a ratio of 3:1:1 (*v*/*v*) was filled in the trays. The growth chamber was maintained at 25 °C using a Daikin air conditioner 24,000 btu (model: FTXM71XVMV, Thailand), with relative humidity controlled at 70 ± 10%, corresponding to a relative humidity range of 60–80%, using a humidifier (model: HQJS150, Haoqi, Zhongshan, China). The seedlings were irrigated every two days with water and once a week with 50 mL of ½ MS solution (Table 5). Each experimental treatment consisted of six trays, and each tray contained 6 plants grown under identical conditions. For physiological measurements, 36 plants from each treatment were measured, and the mean value per tray was used for statistical analysis. The values of plant physiological parameters were calculated per square meter of growing, on a per-tray basis (n = 6 replications). For essential oil extraction, plant material from 36 plants within each treatment was divided into 3 portions, and the extraction was performed separately for each treatment. Subsequently, the essential oil from each treatment was combined and analyzed in triplicate to determine its chemical composition.

**Figure 3:**
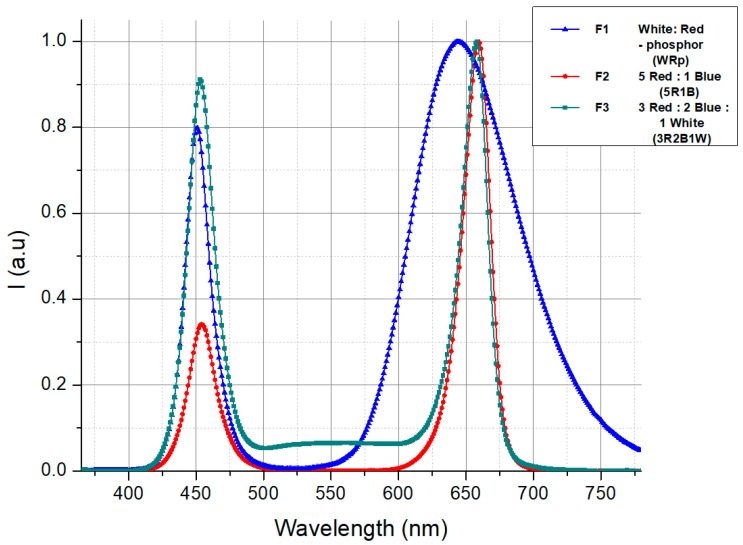
Normalized spectral distribution of LED lighting.

Then, a multiple spectral LED lighting experiment was held during 11 to more than 14 weeks from 11 March 2024 to 20 June 2024. Three LED treatments conducted by combinations of different light spectra, including red (R), white (W), and blue (B), were measured using the CL-500A Illuminance Spectrophotometer, Konica Minolta, Tokyo 100-7015, Japan. F1-treatment consists of a W-LED and R-phosphor; F2-treatment is a combination of semiconductor LEDs with narrow bandwidths at 460 nm (B-LED) and 660 nm (R-LED); F3-treatment is optically configured by semiconductor B- and R-LEDs, which were augmented with a certain small number of W-phosphor-converted LEDs. The LED light intensity was maintained at 2500 lux ± 100 lux (~ 199–208 µmol·m^−2^·s^−1^) measured using T-10A Illuminance Meter, Konica Minolta, Tokyo 100-7015, Japan, which caused no photodamage or heat stress to the plants [64], and was applied continuously 16 h/day, starting before sunrise and extending after sunset (Table 6 and Figure 3).

To compare the spectral ratios between the three treatments, their common point is that they all have the highest amounts of R and B spectra, and almost no UV spectra. The difference is that treatment F1 contains the highest amount of Fr and the lowest amount of B, compared to the other two treatments. F2 has almost no G- and Fr-light, but the highest amount of R. Treatment F3 contains the highest amount of B and G, compared to F1 and F2, almost no Fr, and a slightly higher UV.

**Figure 4:**
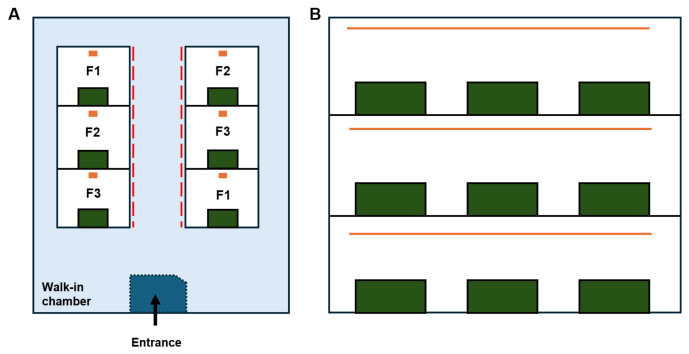
Schematic experimental design for the cultivation of *Tagetes erecta*. Note: (**A**) A side view of a rack with three chambers; three LED treatments, including F1, F2, and F3, were randomly arranged in each rack; green boxes indicated African marigold plant trays, orange rectangle dots indicated different LED sources; red long dash line indicated a light blocker for stopping light interference. (**B**) A front view of the rack; three African marigold trays were placed randomly on each chamber along the LED sources.

**Figure 5:**
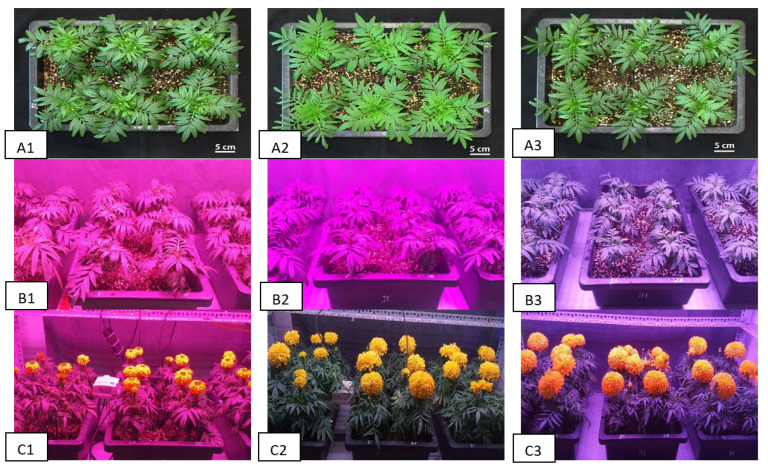
*Tagetes erecta* cultivated under different light conditions. Note: (**A1**–**A3**,**B1**–**B3**): *T. erecta* at the stage of vegetative growth; (**C1**–**C3**): *T. erecta* at harvest; (**A1**,**B1**,**C1**): Plants under treatment F1; (**A2**,**B2**,**C2**): Plants under treatment F2; (**A3**,**B3**,**C3**): Plants under treatment F3.

The T5-type LED lamps with a length of 1.2 m were installed above the growth chamber at an approximate distance of 70 cm from the chamber floor to ensure uniform light distribution and spectral blending. The lamps were centrally positioned and aligned longitudinally along the growth chamber. Each treatment was conducted in two chambers and included 36 individual plants distributed across six trays. Plants in each chamber were isolated using black sheets to prevent interference from other light sources (Figure 4 and Figure 5). The aerial parts of the African marigolds were harvested at the time that at least one flower per plant was fully blooming, for further analysis and evaluation.

### 4.2. Essential Oil Isolation

Each African marigold sample, consisting of 1.1–1.5 kg of aerial biomass, was shredded and subjected to hydrodistillation for 3.5 h using a Clevenger-type apparatus [65]. The obtained essential oil was then separated and stored at –5 °C for subsequent analysis.

### 4.3. Essential Oil GC-MS and GC-FID Analysis

Essential oils were analyzed by GC/MS-FID using an Agilent 7890A GC system coupled with a 5975C Mass Selective Detector. Separation was performed on an HP-5MS fused silica capillary column (60 m × 0.25 mm i.d., 0.25 μm film thickness). Helium was used as the carrier gas at a flow rate of 1.0 mL/min. The injector was set at 250 °C, with a 1 μL injection volume in split mode (1:100). The oven program started at 60 °C and was increased to 260 °C at 4 °C/min. Detector temperatures were maintained at 280 °C. For MS analysis, conditions included an interface temperature of 280 °C, electron ionization (EI) at 70 eV, a scan rate of 4.0 scans/s, and a mass range of 35–450 Da. FID analysis was conducted under identical chromatographic conditions, with the detector temperature also set at 250 °C. Constituents were identified by comparing their relative retention indices (determined by co-injection with a homologous series of *n*-alkanes, C7–C30) and mass spectral fragmentation patterns with reference libraries (NIST08, Wiley09, and HPCH1607) [66,67,68]. Data was processed using *MassFinder* 4.0. Relative concentrations were calculated from FID peak areas without standardization.

### 4.4. Tested Microbial Strains

The antimicrobial activity of the essential oils was evaluated against three Gram-positive bacteria (*Staphylococcus aureus* ATCC 13709, *Bacillus subtilis* ATCC 6633, and *Lactobacillus fermentum* VTCC N4), three Gram-negative bacteria (*Salmonella enterica* VTCC, *Escherichia coli* ATCC 25922, and *Pseudomonas aeruginosa* ATCC 15442), and one yeast strain (*Candida albicans* ATCC 10231). ATCC strains were obtained from the American Type Culture Collection, while VTCC strains were provided by the Vietnam Type Culture Collection, Institute of Microbiology and Biotechnology, Vietnam National University, Hanoi.

### 4.5. Screening of Antimicrobial Activity of Essential Oil

The minimum inhibitory concentration (MIC) and half-maximal inhibitory concentration (IC_50_) of the essential oils were determined in triplicate using the broth microdilution method [69,70]. Stock solutions were prepared in dimethyl sulfoxide (DMSO) and serially diluted with sterile distilled water to yield concentrations ranging from 16,000 to 1000 μg/mL (serial dilutions include five concentrations: 16,000, 8000, 4000, 2000, and 1000 μg/mL). Dilutions were prepared in microtubes and transferred into 96-well microplates. Bacterial strains were cultured in double-strength Mueller–Hinton broth or double-strength tryptic soy broth, while fungal strains were grown in double-strength Sabouraud dextrose broth. Microbial suspensions were adjusted to 5 × 10^5^ CFU/mL for bacteria and 1 × 10^3^ CFU/mL for fungi. Negative controls contained culture medium without essential oil dilutions and microorganisms, and positive controls consisted of culture medium and microorganisms. Plates were incubated at 37 °C for 24 h, and MIC values were defined as the lowest concentration that completely inhibited visible microbial growth. IC_50_ values were calculated from the percentage of growth inhibition, based on turbidity measurements recorded with an EPOCH2C spectrophotometer (BioTek Instruments, Winooski, VT, USA). Data were analyzed using *Raw Data* software (Intercity Business Park Mechelen Noord, Mechelen, Belgium) according to the following equations:

(1) $$\%\;inhibition=\frac{{OD}_{control\left(+\right)}-\;{OD}_{test\;agent}}{{OD}_{control\left(+\right)}-{OD}_{control\left(-\right)}}\times 100\%$$

(2) $${IC}_{50}={High}_{Conc\;}-\frac{{(High}_{Inh\%\;}-50\%)\;({High}_{Conc}-{Low}_{Conc})}{\left({High}_{Inh\%}-{Low}_{Inh\%}\right)}$$

where OD_control (+)_: Optical density of positive control sample (cells in medium without antimicrobial agent); OD_test agent_: Optical density of test samples (each sample corresponds to a specific, known concentration of the antimicrobial agent); and OD_control (−)_: Optical density of negative control sample (culture medium without cells). High_Conc_/Low_Conc_: High and low concentrations of the test agent, respectively; High_Inh%_/Low_Inh%_: Percentage of microbial growth inhibition at high and low concentrations, respectively.

Reference materials: Ampicillin was used as a reference for Gram (+) bacteria, with IC_50_ and MIC values of 0.02–3.62 µg/mL and 0.125–32.0 µg/mL, respectively. Cefotaxime served as the reference for Gram (–) bacteria, showing IC_50_ values of 0.07–4.34 µg/mL and MIC values of 0.5–32.0 µg/mL. For fungal strains, nystatin was employed, exhibiting an IC_50_ of 1.32 µg/mL and an MIC of 8.0 µg/mL.

### 4.6. Statistical Analysis

Data of African marigolds were evaluated using a single-factor completely randomized ANOVA to assess the effects of different lighting treatments. When significant differences were observed, mean separation was performed using the least significant difference (LSD) test at *p* ≤ 0.05. All statistical analyses were conducted with IRRISTAT version 5.0 (International Rice Research Institute, Philippines). Pearson correlation analysis was performed using SPSS ver. 27.0.1 (IBM SPSS Statistics) to examine the relationship between the R:B ratio, essential oil concentration, (*E*)-*β*-ocimene concentration, and antimicrobial activity. PAC analysis was performed using software PCA & HCPC Analysis ver.1.0 (Nguyen, Trung Duc, https://ntducphd.shinyapps.io/pca_plus/ (accessed on 22 April 2026)) to clarify overall trends in the dataset and highlight differences among treatments, as well as the main factors contributing to biomass and essential oil yield.

## 5. Conclusions

Light quality plays a pivotal role in regulating both vegetative and reproductive growth in plants. In this study, a high proportion of R-light combined with a specific B spectrum was particularly effective in maximizing plant performance, significantly increasing above-ground biomass, enhancing flower yield, and producing heavier flowers. This spectral combination also stimulated essential oil biosynthesis and accumulation in African marigolds, while conferring stronger antibacterial activity against *E. coli*. In contrast, phosphor-coated W LED light, which contains a relatively high fraction of Fr-light, promoted earlier flowering and increased flower number. This treatment also resulted in a higher concentration of oxygenated monoterpenes in the essential oils, which might exhibit the strongest antimicrobial activity against *S. aureus*, *S. enterica*, and *C. albicans*. Overall, these findings demonstrate the potential of spectral optimization to enhance crop productivity and phytochemical quality in controlled environments. Tailored lighting regimes can be strategically designed to achieve specific production goals—such as improving flower morphology, increasing yield, or enhancing essential oil content and bioactivity—thereby enabling precise control of plant development and secondary metabolite accumulation in controlled-environment agriculture.

## Figures and Tables

**Table 1:** Biomass and essential oil yield of *Tagetes erecta* cultivated under different light conditions.

Treatment	Fresh Yield of Shoot (g/m^2^)	Water Content (%)	Dry Yield of Shoot (g/m^2^)	Essential Oil Content (% *w*/*w*, dry)	Essential Oil Yield (mg/m^2^)
F1	1157 ± 170 ^b^	89.2 ±0.095 ^c^	125 ± 18.4 ^b^	0.1541 ± 0.0101 ^c^	222 ± 32.7 ^c^
F2	1586 ± 164 ^a^	90.2 ± 0.124 ^a^	155 ± 16.0 ^a^	0.2285 ± 0.0155 ^a^	307 ± 31.7 ^a^
F3	1568 ± 123 ^a^	90.0 ± 0.131 ^b^	157 ± 12.3 ^a^	0.1974 ± 0.0126 ^b^	252 ± 19.8 ^b^

Note: Mean values followed by the same letter within a column are not statistically different at a 0.05 significance level (n = 6). Statistical analyses were performed using IRRISTAT ver. 5.0 (International Rice Research Institute, Laguna, Philippines).

**Table 2:** Composition of essential oils of *Tagetes erecta* cultivated under different light conditions.

Compounds ^a^	RI ^b^	F1 ^c^	F2 ^c^	F3 ^c^
α-Pinene	940	0.2	0.2	0.2
Sabinene	980	0.4	0.4	0.4
Myrcene	993	3.3	2.3	2.5
(3*Z*)-Hexenyl acetate	1006	0.1	0.2	0.1
Limonene	1035	5.2	5.5	5.7
(*Z*)-β-Ocimene	1039	3.2	3.5	3.8
(*E*)-β-Ocimene	1050	22.9	27.3	28.8
γ-Terpinene	1064	Tr	0.1	Tr
Terpinolene	1095	4.5	5.5	5.1
Linalool	1102	0.8	0.6	0.5
1,3,8-*p*-Menthatriene	1119	0.3	0.3	0.3
(*Z*)-Myroxide	1134	0.2	0.1	0.1
(*E*)-Myroxide	1145	20.6	15.0	13.9
*iso*-Menthol	1180	0.2	0.1	0.1
Terpinen-4-ol	1187	0.1	0.1	0.1
(3*Z*)-Hexenyl butanoate	1190	0.5	0.9	0.8
*p*-Cymen-8-ol	1192	0.2	Tr	Tr
*p*-Methylacetophenone	1193	0.2	Tr	Tr
Octyl acetate	1211	Tr	Tr	0.3
2-Phenylethyl acetate	1263	2.7	2.3	2.3
Piperitone	1266	7.3	8.7	9.6
3-Thujyl acetate	1296	4.0	4.8	5.1
Indole	1302	1.2	1.1	1.1
Piperitenone	1354	6.2	5.4	4.3
Piperitenone oxide	1378	2.6	1.7	2.1
α-Santalene	1433	Tr	Tr	0.2
(*E*)-β-Caryophyllene	1439	2.7	3.4	2.9
(Z)-β-Farnesene	1461	0.5	0.5	0.5
Germacrene D	1500	1.5	1.6	1.4
Bicyclogermacrene	1516	0.9	1.3	1.1
(*E*)-Nerolidol	1570	0.3	0.4	0.3
Spathulenol	1598	0.1	0.1	Tr
Caryophyllene oxide	1606	0.2	0.3	0.2
Neophytadiene	1842	0.6	0.8	0.9
Total		93.7	94.5	94.7
Monoterpene hydrocarbons		40.2	45.1	46.8
Oxygenated monoterpenes		42.0	36.5	35.8
Sesquiterpene hydrocarbons		5.6	6.8	6.1
Oxygenated sesquiterpenes		0.6	0.8	0.5
Diterpene hydrocarbons		0.6	0.8	0.9
Benzenoids		2.9	2.3	2.3
Oxylipins		0.6	1.1	1.2
Others		1.2	1.1	1.1
Number of compounds quantified		31	30	30

Note: ^a^ Order of compounds eluted on the HP-5MS column; ^b^ RI: retention index of compounds on the HP-5MS column; ^c^ Standard deviations were insignificant and excluded from the Table to avoid congestion (n = 3); Tr: Trace (concentration < 0.1%).

**Table 3:** Antimicrobial activity of essential oils of *Tagetes erecta* cultivated under different light conditions.

Treatments	Parameters	The Concentration of Essential Oil Inhibiting the Tested Microorganisms (µg/mL)						
		Gram (+) Bacteria			Gram (−) Bacteria			Yeast
		*Staphylococcus aureus*	*Bacillus subtilis*	*Lactobacillus fermentum*	*Salmonella enterica*	*Escherichia coli*	*Pseudomonas aeruginosa*	*Candida albicans*
F1	IC_50_	5134 ± 267	>16,000	>16,000	13,818 ± 610	6469 ± 320	6536 ± 341	4500 ± 201
	MIC	8000	>16,000	>16,000	>16,000	16,000	>16,000	8000
F2	IC_50_	5183 ± 278	>16,000	12,000 ± 579	>16,000	4774 ± 257	8453 ± 414	4758 ± 225
	MIC	8000	>16,000	>16,000	>16,000	8000	>16,000	8000
F3	IC_50_	9391 ± 476	>16,000	>16,000	16,000 ± 577	13,333 ± 569	7571 ± 377	>16,000
	MIC	>16,000	>16,000	>16,000	>16,000	>16,000	>16,000	>16,000
Ampicillin	IC_50_	0.02 ± 0.005	3.62 ± 0.15	1.03 ± 0.07				
	MIC	0.125 ± 0.0	32 ± 0.0	32 ± 0.0				
Cefotaxime	IC_50_				0.43 ± 0.05	0.007 ± 0.002	4.34 ± 0.15	
	MIC				32 ± 0.0	0.5 ± 0.0	8 ± 0.0	
Nystatin	IC_50_							1.32 ± 0.05
	MIC							8 ± 0.0

**Table 4:** Correlation coefficients between R- to B-light ratio, *Tagetes erecta* essential oils, and their antimicrobial activities.

Main Constituents	R:B Spectral Ratio	Essential Oil Concentration	(*E*)-*β*-Ocimene Concentration	*Staphylococcus aureus*	*Bacillus subtilis*	*Lactobacillus fermentum*	*Salmonella enterica*	*Escherichia coli*	*Pseudomonas aeruginosa*	*Candida albicans*
R:B spectral ratio		−0.418	−0.894	−0.947	.^a^	−0.186	−0.758	−0.866	−0.373	−0.950
Essential oil concentration			0.780	0.104	.^a^	−0.815	0.909	−0.094	0.999 *	0.114
(*E*)-*β*-Ocimene concentration				0.704	.^a^	−0.273	0.970	0.550	0.749	0.711

Note: * Correlation is significant at the 0.05 level (2-tailed). ^a^ Cannot be computed because at least one of the variables is constant. Pearson correlation analysis was performed using SPSS ver. 27.0.1 (IBM SPSS Statistics).

**Table 5:** MS solution used in the cultivation of *Tagetes erecta*.

Nutrients	Components	Concentration (µM)
Macro-nutrients	Nitrogen	298.95
	Phosphorous	62.00
	Potassium	155.95
	Calcium	17.00
	Magnesium	7.50
	Sulfur	13.55
Micro-nutrients	Iron	0.50
	Manganese	5.00
	Zinc	0.50
	Boron	0.50
	Copper	0.05
	Molybdenum	0.50
	Cobalt	0.05

**Table 6:** Light conditions in the cultivation of *Tagetes erecta*.

Treatments	F1	F2	F3
LED ratio	White: Red-phosphor (WRp)	5 Red:1 Blue (5R1B)	3 Red:2 Blue:1 White (3R2B1W)
Spectral distribution (%) {(UV < 400 nm):(B~400–500 nm):(G~501–600 nm):(R~601–700 nm):(Fr > 700 nm)}	0.06:17.33:5.88:64.28:12.45	0.07:25.82:0.52:73.45:0.14	0.09:42.05:10.22:47.30:0.34
Total of B & R (%)	81.61	99.27	89.35
The reduced ratio of R to B	3.71:1	2.84:1	1.12:1
Duration (h/day)	16	16	16
Lighting time	6:00–22:00	6:00–22:00	6:00–22:00
Light intensity (µmol·m^−2^·s^−1^)	206 ± 8.2	208 ± 8.3	199 ± 8.0
Total daily light (mol·m^−2^·d^−1^)	11.866 ± 0.472	11.981 ± 0.478	11.462 ± 0.461

## Data Availability

All data are available in this publication.

## Abbreviations

The following abbreviations are used in this manuscript:

B	Blue
DLI	Daily light integral
Fr	Far red
G	Green
IC_50_	Half-maximal inhibitory concentration
MIC	Minimum inhibitory concentration
PCA	Principal component analysis
R	Red
Rp	Red-phosphor
Tr	Trace
UV	Ultra violet
W	White
